# An Interesting Letter

**Published:** 1871-03

**Authors:** 


### AN INTERESTING LETTER.

We call attention to the following letter, taken from the "Journal of the Gynæcological Society of Boston." We have before endorsed the course of Drs. Storer and Sullivan. We go further. We believe the profession endorse their action, and commend the Gynæcological Society for its prompt assumption of all the responsibility of their action. We beg the profession everywhere to keep constantly before them this fact—we would impress it upon them—that individuals must be sustained, at home and abroad, by the profession, when they place themselves upon the principles of the law in opposition to disorganizers and violators of ethical rule. Errors so grave cannot be apologized for, or ignored, without giving "aid and comfort" to the enemy, and by rebound, casting reproach and contempt upon those who seek to uphold and dignify the law. If the profession is not for them, it is against them. There can be no neutral ground upon which the good and bad may stand. The paths of wrong and right never come together. To encourage violations of ethical principles is not only destructive to those principles, and to medical organization, but it is criminal. Criminal, because evil-doers are furnished a plea for the justification of their deeds; wrong, given a weapon to assassinate the right, and the cause of science diverted from its holy mission of ministering to the afflictions of suffering humanity. Establish the principle that violators must feel the weight of the penalties of violated law; then, of necessity, another principle must follow to make the punishment an adequate remedy—to-wit: that the advocates and supporters of disorganizers and violators of law, must either refuse to recognize them professionally, or forfeit all claims to the benefits and privileges of the profession.

"I have seen, through the 'Journal,' that you and Dr. Sullivan have been censured by the Councillors of the Massachusetts Medical Society for entering a protest against the admission of the delegates of that Society to seats at the last meeting of the American Medical Association. I am pained to see that, while the Councillors have acquitted you and Dr. Sullivan, upon ethical grounds, in justification of your action, they yet go behind that action to rebuke your motives. I am rejoiced, however, to know that your action, morally and professionally, has been sustained both by the Massachusetts Medical Society and by the Gynæcological Society, as well as by the action of the American Medical Association.

"It is very clear that no one can, with any show of justice, assail the motives of another, when the act objected to is declared to be based and founded upon the principles of right, and to have received the endorsement of the highest tribunals. Only when the act is wrong, can the motives inducing the act be liable to be suspected to be also wrong. With the profession at large, the motives, whatever they may be, influencing the members of the Massachusetts Medical Society or those of the Gynæcological Society, have nothing to do. Professional men at a distance, untrammelled, must consider and arrive at conclusions from facts.

"The facts show who maintain and support principle or ethics, and who depart from either or both. And each individual member of the profession, in vindication of his private as well as professional character, must support the truth. The question with the profession is this: Did Drs. Storer and Sullivan do their duty as true representatives of a common profession and as members of local societies, in offering their protest against the recognition of the delegates of the Massachusetts Medical Society, upon the ground of irregularities in that Society, as entitled to seats in the American Medical Association? To this only one reply is made. Both the Massachusetts Medical Society and the American Medical Association respond that they did. Here that question must and will, forever rest.

"The next question is, Did the Gynæcological Society do right in instructing Drs. Storer and Sullivan to offer the above-mentioned protest to the American Medical Association? We answer: The various rulings and decisions of the American Medical Association will and do sustain them. And they will not only be sustained by the Association, its constitution and code of ethics, but by the principles governing gentlemen of honor, in assuming all the responsibility of the protest offered by Drs. Storer and Sullivan, and declaring that the attempt to censure these gentlemen is an insult to themselves, which should demand a trial by the Massachusetts Medical Society.

"I assert that the 'conditions imposed' by the American Medical Association upon the Massachusetts Medical Society, with reference to future representation, were not 'ill-considered and unwarranted,' but were, on the contrary, well-timed and well-merited. The 'condition' imposed was to 'purge out irregular practitioners' from its body. These conditions were so just, and the cause for action so flagrant and notorious, that even the Massachusetts Medical Society, by resolution, did purge itself of its irregular members, thereby admitting the justness of the charge. No 'formal representation' to the American Medical Association, 'with a view of procuring a reconsideration of its action,' will weigh one iota with the Association, until the Massachusetts Medical Society shall acknowledge its fault, and promise strict adhesion to ethical law in the future. 'A party under sentence of non-representation,' or of excommunication from that Association, can only be restored to fellowship and recognition by repentance, acknowledgment, and reconciliation of ethical violation. The future holds in reservation no good to those who would defy the Association, and at the same time seek to maintain connection with it.

"All honorable men of the profession, wherever they may live, must adhere to the letter of the ethics; and all who do must be sustained, or what little honor is left in the profession will be lost."

